# Aptamer-based assay for rapid detection, surveillance, and screening of pathogenic *Leptospira* in water samples

**DOI:** 10.1038/s41598-023-40120-w

**Published:** 2023-08-17

**Authors:** Archana Vishwakarma, Yogesan Meganathan, Mohandass Ramya

**Affiliations:** Department of Genetic Engineering, Faculty of Engineering and Technology, School of Bioengineering, SRM Institute of Science and Technology, SRM Nagar, Kattankulathur, Kanchipuram, Chennai, Tamil Nadu 603203 India

**Keywords:** Biotechnology, Molecular biology, Health care

## Abstract

Leptospirosis is a potentially fatal waterborne infection caused by *Leptospira interrogans*, impacting both humans and animals in tropical regions. However, current diagnostic methods for detecting pathogenic *Leptospira* have sensitivity, cost, and time limitations. Therefore, there is a critical need for a rapid, sensitive, and cost-effective detection method. This study presents the development of an aptamer-based assay for pathogenic *Leptospira* detection. Aptamers targeting *Leptospira* were generated using the SELEX method and screened for binding affinity with major Leptospiral outer membrane proteins through in silico analysis. The aptamer with the highest binding affinity was selected for further evaluation. To enable visual detection, the aptamer was conjugated to gold nanoparticles (AuNPs), resulting in a colorimetric response in the presence of *L. interrogans*. The aptamer-AuNP-based colorimetric assay exhibited a detection limit of 57 CFU/mL and demonstrated high specificity and reproducibility in detecting pathogenic *Leptospira* in water samples. This aptamer-based assay represents a significant advancement in leptospirosis diagnostics, offering a rapid, sensitive, and cost-effective approach for detecting pathogenic *Leptospira*. Its potential for epidemiological applications, such as outbreak source identification and improved prevention, diagnosis, and treatment, particularly in resource-limited settings, highlights its importance in addressing the challenges associated with leptospirosis.

## Introduction

Infection with pathogenic *Leptospira* species causes leptospirosis, a widespread waterborne zoonotic disease. Leptospires are spirochetes, and the genus *Leptospira* includes both saprophytic and pathogenic species. Infection with *Leptospira* affects both humans and animals. Treating this life-threatening but curable disease relies on timely diagnosis^1^. However, the initial symptoms of leptospirosis can be easily confused with those of other diseases, such as dengue and malaria, making accurate diagnosis difficult^2^. As a result, misdiagnosis/late diagnosis leads to the progression of a more severe condition known as Weil's disease, which includes kidney or liver failure, respiratory distress, meningitis, and, eventually, death^3^. The Centers for Disease Control and Prevention (CDC, USA), and World Health Organization (WHO, Switzerland) have classified it as an emerging or re-emerging infectious disease^4^.

*Leptospira* is visualized using dark field microscopy (DFM). It requires a minimum of 10^4^ Leptospires/mL. It is neither sensitive nor specific enough to be used as a diagnostic technique^5^. Currently, most diagnostic methods rely on serology. The microscopic agglutination test (MAT) is the reference test for antibody detection and necessitates the maintenance of live *Leptospira* cultures. It is done by incubating patient serum with various *Leptospira* serovars. The serovar that reacts with patient serum is the infecting serovar. However, despite being a reference test, MAT lacks specificity^6^. Other serology-based tests include IgM ELISA^7^, IgM dipstick^8^, IgM dot ELISA^9^, slide agglutination test^10^, and indirect agglutination test^11^. While these are simpler to perform than MAT, their diagnostic accuracies have not been fully established. Furthermore, molecular technologies such as PCR and real-time PCR can identify leptospirosis with surface biomarkers such as *Lipl32*^12^ but are not cost-effective and cannot detect the disease at an early stage^3^.

In recent years, aptamers, which are single-stranded nucleotides, have emerged as a promising biorecognition element for pathogen and small molecule detection due to their high specificity and affinity for their targets. The Systematic Evolution of Ligands by Exponential Enrichment (SELEX) method is commonly used to generate aptamers by exposing naïve DNA aptamer libraries to the target. Aptamers offer several advantages over traditional antibodies, including superior specificity and sensitivity, a broader target range, lower production costs, and longer shelf life^13,14^.

In order to address the challenges associated with *Leptospira* detection, this study aims to present a highly effective aptamer-based assay for the rapid detection of pathogenic *Leptospira*. Using the SELEX method, aptamers specifically targeting *Leptospira* were generated. These aptamers were then screened for their binding affinity with major Leptospiral outer membrane proteins using HDOCK, an in silico docking tool. The aptamer exhibiting the highest binding affinity was further validated using a fluorescence assay, with a determined dissociation constant (K_d_) value of 133.13 nM.

**Figure 1:**
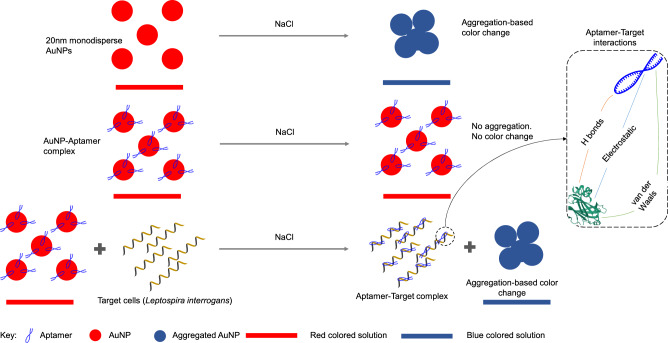
Illustration of the AuNP-Aptamer colorimetric approach used to detect *L. interrogans*.

The use of aptamers as biosensors has proven successful in detecting various pathogens and environmental contaminants. Notably, aptamers have demonstrated their effectiveness in detecting Gram-positive bacteria such as *Staphylococcus aureus*^15^, *Streptococcus pneumoniae*^16^, and *Listeria monocytogenes*^17^. They have also shown efficacy in detecting Gram-negative bacteria, including *Salmonella spp*.^18,19^, *Shigella sonnei*^20^, *Escherichia coli* O157^21^, *Vibrio cholerae*^12^, *V. parahaemolyticus*^22^, and *Pseudomonas aeruginosa*^23^. Furthermore, aptamers have also been utilized to detect the parasitic pathogen *Plasmodium falciparum*^24^. Expanding upon these notable accomplishments, our study represents a significant advancement in the field by reporting the application of aptamers for detecting leptospirosis^25^. We used the whole-cell SELEX approach to generate cell-specific aptamers with high affinity and specificity against *L. interrogans*. Our research paper describes a sensitive assay for the detection of *L. interrogans* using a cell-specific aptamer and gold nanoparticles (Fig. 1). The developed assay enables the detection of *L. interrogans* at low concentrations in a 96-well-plate-based assay format, with no cross-reaction with other bacterial genera. To underscore the practical implications of our findings, we successfully applied the developed assay to screen water samples collected from the Chengalpattu district in Tamil Nadu, India. This highlights the potential for our approach to be deployed in real-world scenarios, aiding in the early detection and surveillance of leptospirosis outbreaks, particularly in regions susceptible to this waterborne disease.

## Materials and methods

### Bacterial strains and culture media

Three pathogenic serovars of *Leptospira interrogans* namely Canicola, Autumnalis, and Pomona, were obtained from Dr. K. Natarajaseenivasan at the Bharathidasan University in Tiruchirappalli, Tamil Nadu, India. All strains were cultured using *Leptospira* Medium Base (Himedia M1009) supplemented with *Leptospira* Enrichment (Himedia FD066), and 5-Fluorouracil (5 mg/mL) was used as the antibiotic to suppress the growth of other bacteria. The cultures were incubated at 28 °C for 14 days for visible growth. Inoculum of 10% volume is used for sub culturing the strains every 14 days. *E. coli* and *P. aeruginosa* were grown in Luria-Bertani (LB) broth, while *S. flexneri* (MTCC 1457) and *V. cholerae* (MTCC 3706) were grown in trypticase soy broth and incubated at 37 °C. Due to the pathogenic nature of the organisms used in this study, all experiments were performed in a biosafety cabinet level II while adhering to safety protocols to minimize the risk of exposure and prevent potential transmission.

### Cell-SELEX procedure

#### Preparation of single-stranded DNA (ssDNA) library

We utilized an 81 bp oligonucleotide single-stranded DNA library comprising a randomized middle region of 45 nucleotides flanked by primer binding regions. The specific sequence of the ssDNA library was 5′- GAGTATCCAGACGCAGCA (N45) TGGACAGGCTTAGTCGGT -3′. Amplification of the template was carried out using 10 μL of the 100 μM ssDNA library, along with the forward primer 5′-GAGTATCCAGACGCAGCA-3′ and the reverse primer 5′-biotin-ACCGACTAAGCCTGTCCA-3′.

To prevent rehybridization of the single-stranded DNA library, the diluted template underwent heat treatment at 95 °C for 10 min, followed by immediate cooling in an ice bath. The single-stranded DNA oligos were incubated overnight at 4 °C to attain their desired 3D conformations.

#### In vitro* cell-SELEX*

This study employed a modified microtiter plate-based cell SELEX to select aptamers against *L. interrogans* serovar Autumnalis. Initially, a sterile microtiter plate was functionalized with 2.5 mM Phenylboronic Acid (PBA) in filter-sterile carbonate buffer (pH 9.2) and incubated overnight at 4 °C. Then, 1 × 10^5^ cells were added to the well and incubated at 25 °C for 1 h. The plate was washed thrice with 200 μL Tris-HCl binding buffer (BB) to remove unbound cells. Next, a naïve aptamer library was added to the well and incubated at 25 °C for 1 h. The solution was washed thrice with Tris-HCl binding buffer to remove loosely bound sequences. Elution buffer (10 mM Tris, 0.1 mM EDTA, pH 8.5) was added to the well, and the mixture was allowed to sit for 5 min before it was carefully drawn using a pipette without touching the walls of the microtiter plate. The resulting binder aptamer pool was quantified and stored at − 20 °C. The amplification and partitioning of the binder pool were carried out using a previously described method, and ssDNA was quantified at the end of each round. A total of ten rounds of selection were performed using fresh aliquots of cells. To eliminate non-specific aptamers, three rounds of counter SELEX were conducted against closely related organisms *(E. coli* and *V. cholerae)* at rounds 5 and 8.

### Identification of specific aptamer sequences

The purified PCR products were cloned into *E. coli* TOP10 cells after the final selection round. Transformed *E. coli* cells were randomly chosen to harvest the recombinant plasmids. These plasmids were sequenced using Sanger Sequencing, which yielded 16 sequences. These sequences were screened for their ability to bind with their target using HDOCK for protein–DNA docking (http://hdock.phys.hust.edu.cn/)^26^. The aptamer sequences were docked against six major Outer Membrane Proteins of *L. interrogans* (Lipl32, Lipl21, Lipl41, OMPL1, LigA, LigB)^27^. The secondary structures of the top three binding aptamers were predicted using the Mfold application hosted on the UNAfold Web Server (http://www.unafold.org/mfold/applications/dna-folding-form.php) at 25 °C in 0.05 M NaCl^28^.

### Analysis of binding affinity

The selected aptamer was labelled with a fluorophore to determine its binding affinity with L. interrogans. The single-stranded DNA (ssDNA) aptamer was labelled with fluorescein isothiocyanate (FITC). An increasing concentration of FITC-labelled ssDNA aptamer (0–1000 nM) was added to a constant cell concentration of *L. interrogans* serovar Autumnalis (6 × 10^5^ CFU/mL) for the binding assay. After incubating with FITC-labelled aptamer at room temperature for 15 min with gentle rotation in 500 μL 1X BB, three washes were performed to eliminate the unbound aptamers. The fluorescence emission spectra for binding affinity studies were recorded using a Hitachi-F4700 fluorescence spectrophotometer. Fluorescence microscopy was used to view the aptamer-bound cells (Leica, Germany). A 492 nm laser was used to stimulate the FITC, and a 40 × objective was used to gather the fluorescence data. The assay was repeated with different bacterial populations to evaluate cross-reactivity.

### Aptamer-gold nanoparticle-based colorimetric assay for the detection of *L. interrogans*

A colorimetric detection method was developed to detect *L. interrogans* using a single-stranded DNA (ssDNA) aptamer as the bio-recognition element and 20 nm citrate-capped gold nanoparticles (AuNPs) obtained from Sigma Aldrich (Cat. No. 741965) as the indicator. The aptamer was prepared by denaturing a 50 μL solution of 300 nM aptamer in a 10 mM PBS buffer and then incubated with various concentrations of target cells for 5 min at room temperature. The aptamer-cell mixture was transferred to a 96-well microtiter plate, and 50 μL of 20 nm AuNPs solution was added, followed by a 2-min settling period. Subsequently, 50 μL of 50 mM NaCl was introduced to induce aggregation, and the total volume was adjusted to 150 μL. Visual color changes resulting from different aptamer concentrations in the presence of *L. interrogans* were quantitatively analyzed, and corresponding UV–visible spectra were recorded. Additionally, comprehensive TEM analysis was employed to visualize the morphological evolution of AuNPs upon exposure to the target cells. The entire assay was conducted under controlled conditions, maintaining a pH of 7.4 and a temperature of 25 °C. To ascertain the specificity of the assay, optimization and control experiments were also performed.

### Screening of environmental water samples

A study was designed to investigate the effectiveness of the developed assay for detecting *L. interrogans* in water samples. A total of 25 water samples were collected from Chengalpattu district in Tamil Nadu, India, during July to November 2022. These samples were selected based on their availability and representation of different water sources in the region. Among the collected samples, 16 were obtained from puddles, while the remaining 9 were collected from paddy fields. The water samples were filtered through Whatman ® qualitative filter paper, Grade 1 (Sigma-Aldrich, USA), to remove any debris that might interfere with the assay. The colorimetric assay was carried out using 50 μL of water sample and the same volumes of each component as described in the previous section. Additionally, these samples were screened for *L. interrogans* using a PCR assay targeting the *Lipl32* gene^29^.

## Results

### Selection of ssDNA aptamers against *L. interrogans*

Cell-SELEX was used to select high affinity, single-stranded DNA aptamers that recognize *L. interrogans,* and negative selection was performed using *E. coli, S. flexneri,* and *V. cholerae* to reduce non-specific aptamers. The aptamers directed towards *L. interrogans* were cloned and sequenced after ten rounds of Cell-SELEX. Sequence analysis of all clones revealed that some sequences dominated the pool, and these sequences were investigated further as potential aptamers. Docking studies showed that three aptamers (LAP1, LAP2, and LAP3) outperformed the other aptamers in target recognition. LAP3 5′- TGGCGTTAGAGATACCGGAACCGGTGTCGGGCGTCTGAAGAATCC-3’ (Patent Application No. 202241038353) demonstrated the most substantial interaction with *L. interrogans* based on docking scores and was chosen for the subsequent assays. The detailed results of the aptamer selection process, including sequences of the identified aptamers are available in the Supplementary Data (Supplementary Table 1 and Supplementary Figs. 1, 2 and 3).

### Analysis of binding affinity and secondary structure of LAP3

**Figure 2:**
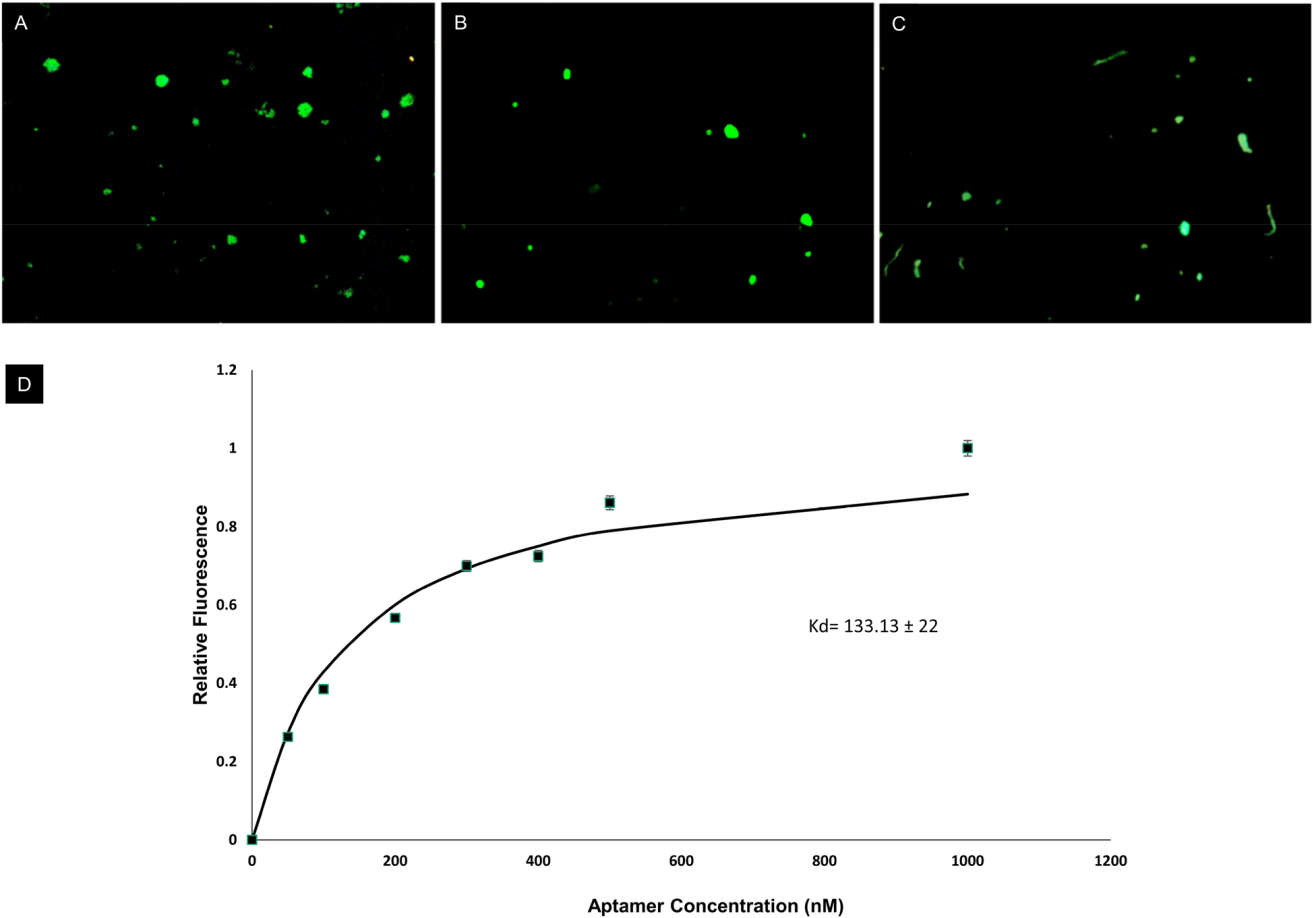
FITC-labeled aptamers bound to different serovars of *L. interrogans* (**A**) Autumnalis, (**B**) Canicola, (**C**) Pomona. (**D**) The binding affinity of selected aptamer LAP3 towards *L. interrogans* using FITC-labeled aptamer.

**Figure 3:**
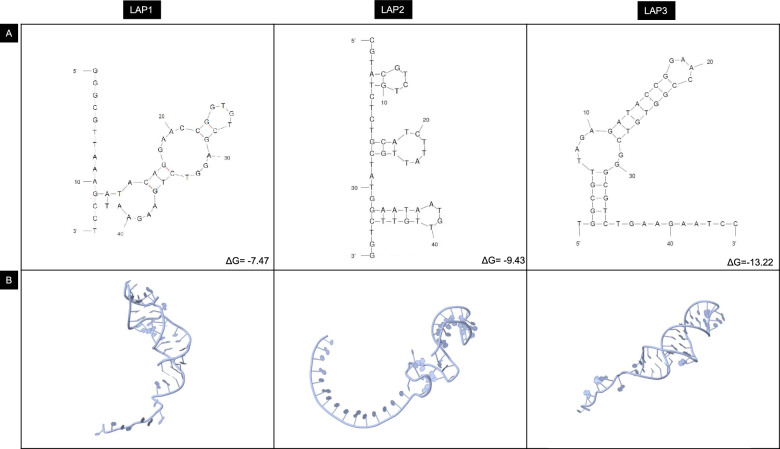
(**A**) Predicted secondary structures of the aptamer sequences showing the lowest free energy. (**B**) Computational prediction of the 3D structures of the aptamers.

The fluorescently labeled aptamers bound to *L. interrogans*, but no binding was observed with any of the other related bacteria used in the study (Fig. 2). The dissociation constant (K_d_) of LAP3 was measured to be 133.13 nM (Fig. 2D). The Mfold Web Server was used to predict the secondary structures of the aptamers based on free energy minimization techniques. The most thermodynamically stable structures were chosen. Analysis revealed a classic stem-loop structure that plays a significant role in the aptamer binding to surface markers on *L. interrogans* spp (Fig. 3).

### Colorimetric detection of *L. interrogans*

**Figure 4:**
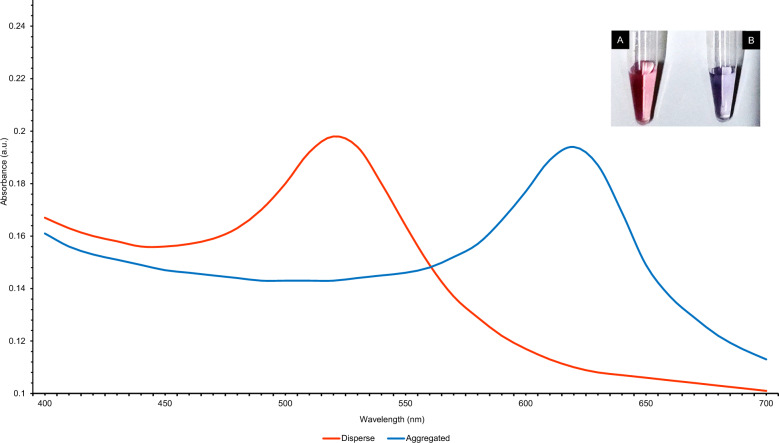
UV–vis absorption spectra of the AuNPs before and after adding target cells. (**A**) AuNPs retained the red color in the presence of 50 mM NaCl and selected aptamer. (**B**) AuNPs solution readily turned blue in the presence of *L. interrogans*.

**Figure 5:**
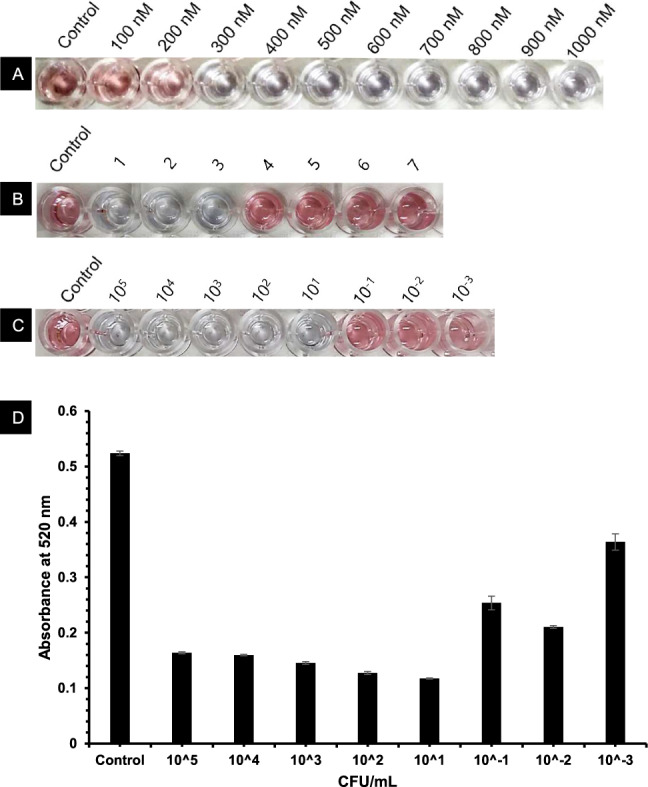
(**A**) Effect of different aptamer concentrations on *L. interrogans* (6 × 10^5^ CFU/mL). (**B**) Visible effect on AuNP in presence of various organisms 1: *L. interrogans* serovar Canicola 2: *L. interrogans* serovar Autumnalis 3: *L. interrogans* serovar Pomona 4: *P. aeruginosa* 5: *E. coli* 6: *V. cholerae* 7. *S. flexneri*. (**C**) Sensitivity of AuNP-based colorimetric assay for *L. interrogans* (6 × 10^5^ to 6 × 10^–3^ CFU/mL). (**D**) UV–Vis data of AuNP-based colorimetric assay for *L. interrogans* (6 × 10^5^ to 6 × 10^–3^ CFU/mL).

**Figure 6:**
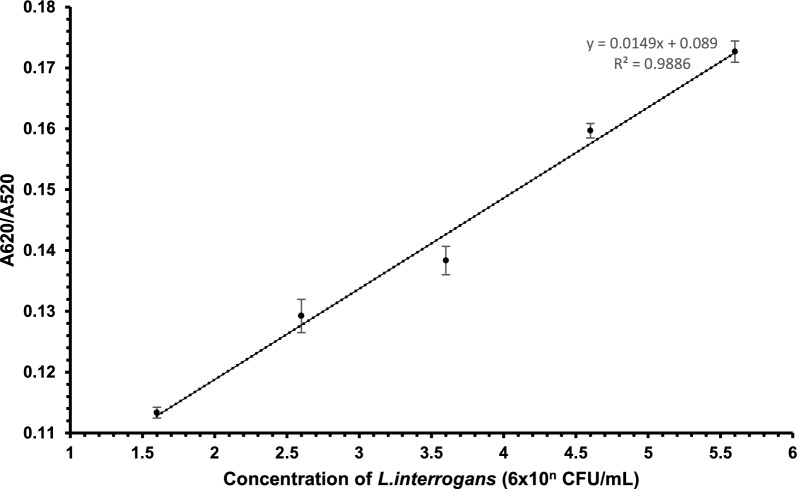
Characterization of AuNP-based colorimetric assay for *L. interrogans*. A linear regression analysis of the assay response to different target concentrations. Each value represents the mean of 3 independent experiments. Error bars represent the standard deviation of the 3 measurements.

**Figure 7:**
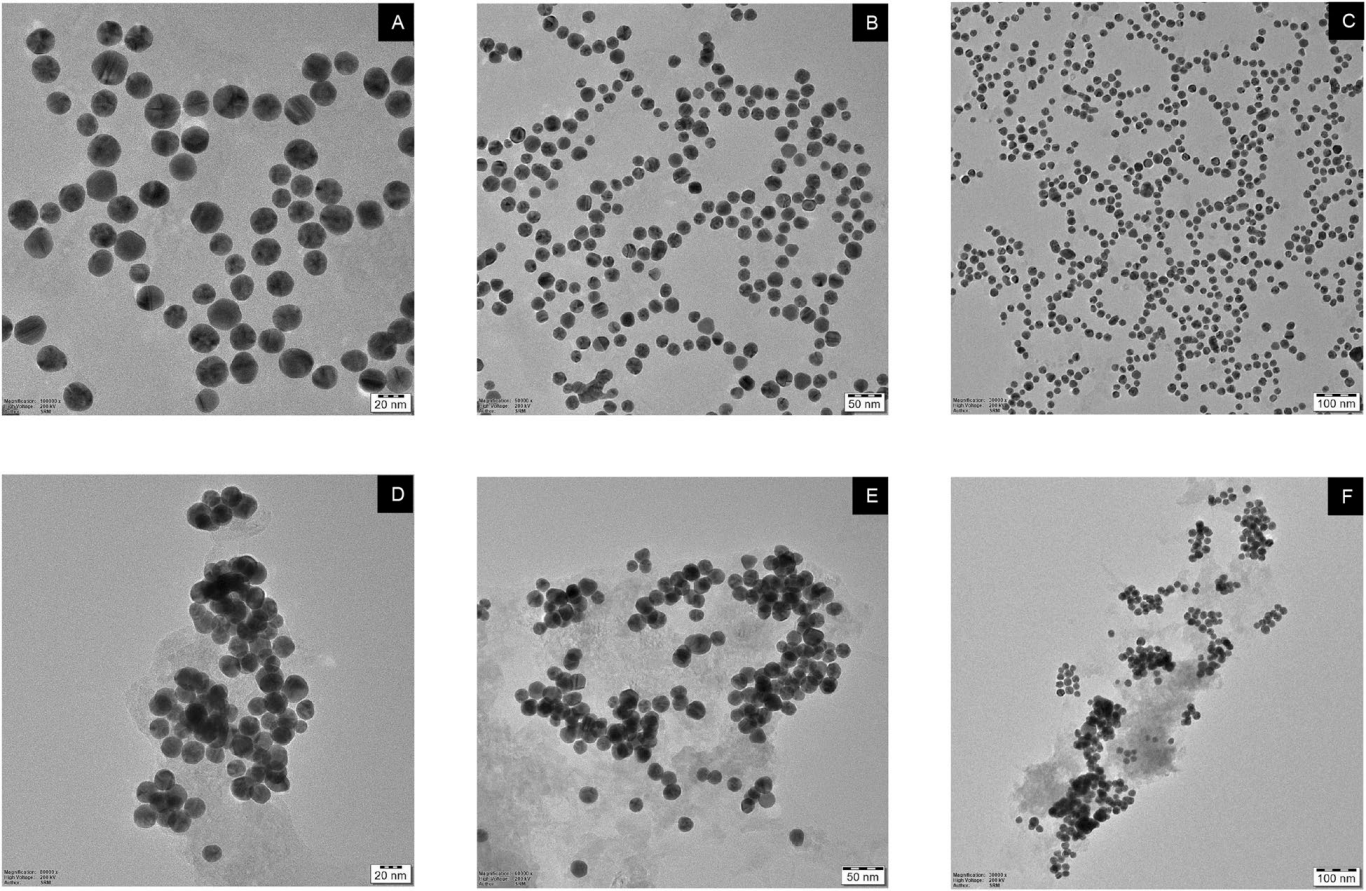
(**A**–**C**) TEM images of the nanoparticles obtained from the solution containing Apt-AuNPs and NaCl. (**D**–**F**) TEM images of the nanoparticles obtained from the solution containing Apt-AuNPs, *L. interrogans*, NaCl.

Gold nanoparticles were used to develop an assay for detecting *L. interrogans* based on color changes caused by aptamer binding and salt-induced aggregation. The absorbance spectra analysis showed that the dispersed gold nanoparticles (AuNPs), characterized by their red hue, exhibited a maximum absorbance peak at 520 nm, a well-established wavelength for dispersed AuNPs^30^. On the other hand, the aggregated AuNPs, which exhibited a blue hue, had a maximum absorbance peak at 620 nm (Fig. 4). The assay was highly selective, with no cross-reactivity observed for closely related bacteria. The sensitivity of the assay was evaluated using different concentrations of *L. interrogans*, with a linear response observed in the concentration range of 6 × 10^5^ to 60 CFU/mL (Fig. 5). The Limit of Detection (LOD) was estimated to be 57 CFU/mL using the equation LOD = 3.3 × standard deviation of the regression line/slope (Fig. 6). TEM analysis confirmed that Apt-AuNP aggregation only occurred in the presence of *L. interrogans* (Fig. 7).

### Prevalence of *L. interrogans* in environmental water samples

The screening results demonstrated that among the 25 water samples examined, the PCR assay detected the presence of *L. interrogans* in 16 samples (64%), whereas the aptamer assay identified *L. interrogans* in 14 samples (56%) (Table 1). Considering the sample size employed in this study, a Fisher's exact test was performed to investigate the potential association between the results of the PCR assay and the aptamer assay for detecting *L. interrogans* in the water samples. The resulting p-value obtained from the test was calculated as 0.0837. Based on this statistical analysis, no statistically significant difference was observed between the PCR and aptamer assays regarding their capability to detect *L. interrogans* in the tested water samples.

**Table 1:** Prevalence of *Leptospira* in different water sources.

S. no.	Water source	PCR Positive	Aptamer assay positive
1	Puddles	12/16	10/16
2	Paddy Fields	4/9	4/9
Total		16/25	14/25

## Discussion

The results presented here demonstrate the successful selection of a high-affinity and specific aptamer (LAP3) against *L. interrogans*. The SELEX process resulted in isolating a highly specific aptamer, LAP3, that selectively binds to *L. interrogans* with high affinity. The Mfold Web Server was used to predict the secondary structures of the aptamers based on free energy minimization techniques. The most thermodynamically stable structures were chosen^28^. The analysis revealed a classic stem-loop structure that plays a significant role in the aptamer binding to surface markers on *L. interrogans* spp^31^. The aptamer could bind to the target cells with a dissociation constant of 133.13 nM and distinguish *L. interrogans* from closely related bacteria, as demonstrated by the lack of a colorimetric response in the presence of non-target bacteria.

The binding of LAP3 to *L. interrogans* was also confirmed using a gold nanoparticle-based colorimetric assay. This colorimetric assay takes advantage of the particle-size dependences of gold nanoparticle surface plasmon resonance (SPR). It has been demonstrated that when particles in a sample interact, the spectra of these nanoparticles change dramatically^32^. At 520 nm, the red-colored AuNPs (20 nm in diameter) display high SPR absorption. In the presence of 50 mM NaCl, AuNPs stabilized at the surface by adsorbed citrate anion aggregate instantaneously and irreversibly. As a result of this aggregation, the SPR absorption shifts to a longer wavelength, resulting in the characteristic red-blue colour change^33^. In the presence of the aptamer LAP3, the AuNPs retained their red colour due to stable binding of the aptamer with the AuNPs. when 50 mM NaCl was added. This interaction between gold and nitrogenous bases thereby stabilizes AuNPs against salt-induced aggregations. However, when *L. interrogans* was present, the AuNPs solution immediately became blue. This change in colour shows that the chosen aptamer forms a tertiary structure with the target cell that lacks affinity to AuNPs, leading to salt-induced aggregation^34^.

The absorbance spectra analysis demonstrated distinct characteristics of the dispersed and aggregated gold nanoparticles (AuNPs). The dispersed AuNPs exhibited a red color and displayed a maximum absorbance peak at 520 nm, consistent with the typical wavelength of dispersed AuNPs. On the other hand, the aggregated AuNPs appeared blue and exhibited a maximum absorbance peak at 620 nm. The detection limit for *L. interrogans* using this assay was as low as 57 CFU/mL. The assay exhibited a linear response within the concentration range 6 × 10^5^ to 60 CFU/mL, indicating its sensitivity and accuracy in detecting varying concentrations of *L. interrogans*. The Apt-AuNP aggregation was monitored using TEM, which confirmed that the color change observed in the assay was due to the aggregation of AuNPs in the presence of *L. interrogans*. The aptamer assay was used to investigate the prevalence of *L. interrogans* in 25 water samples collected from different sources in the Chengalpattu district of Tamil Nadu, India. The PCR assay detected the presence of *L. interrogans* in 16 samples (64%), while the aptamer assay identified *L. interrogans* in 14 samples (56%). The comparable detection rates between the PCR assay and the aptamer assay highlight the potential utility of the aptamer assay as a valuable tool for detecting and identifying leptospiral contamination in water bodies.

## Conclusions

This study employed cell-based SELEX to generate aptamers against *L. interrogans*. The high specificity and sensitivity of the developed assay make it a promising tool for diagnosing leptospirosis. This disease is often difficult to diagnose due to its non-specific symptoms and the lack of reliable diagnostic tests^11^. These features, coupled with the ease of use and low cost, make it a promising candidate for use in resource-limited settings where the disease is most prevalent. The binding affinity of the selected aptamer was confirmed fluorometrically. A nanoparticle-based colorimetric detection system was developed in the microtiter plate format. The lack of cross-reaction of the developed platform with closely-related bacterial species supports its potential application in the detection of *L. interrogans* in environmental samples as well as clinical samples. This colorimetric assay demonstrated high sensitivity and specificity and can be completed in under 15 min using small sample volumes (50 μL). It generates an easy-to-interpret colorimetric output that can be coupled to portable devices like smartphones and tablets, allowing detection even in resource-limited settings. Further efforts are underway to enhance the sensitivity of the aptamer to match or surpass the performance of existing molecular methods, thus addressing the limitations of current techniques while maintaining a high level of target sensitivity. The development of this colorimetric assay for *L. interrogans* detection represents a significant advancement with potential implications for improved diagnosis and treatment of leptospirosis.

### Supplementary Information

Supplementary Information.

## Data Availability

This article contains all relevant data necessary to support the findings presented. We have thoroughly disclosed and included all pertinent data within the manuscript, ensuring transparency and reproducibility. The datasets, methodologies, and analytical procedures employed in this study are described in detail in the Methods section. Any additional information required to reproduce the results and conclusions of this research can be obtained by contacting the corresponding author.
