# Supplementary material for: Aptamer-based assay for rapid detection, surveillance, and screening of pathogenic Leptospira in water samples
Source: Sci Rep. 2023 Aug 17;13:13379. doi: 10.1038/s41598-023-40120-w (PMC10435560; doi:10.1038/s41598-023-40120-w)
Supplement: Supplementary file 1 — Supplementary Information. [file 41598_2023_40120_MOESM1_ESM.pdf]

Supplementary Information

Table 1: Absorbance values of binders from each SELEX round

SELEX Round	Absorbance at 260nm (ng/ul)
1	0.125
2	0.185
3	0.391
4	0.693
5	1.26
6	1.57
7	2.31
8	2.66
9	3.11
10	3.89

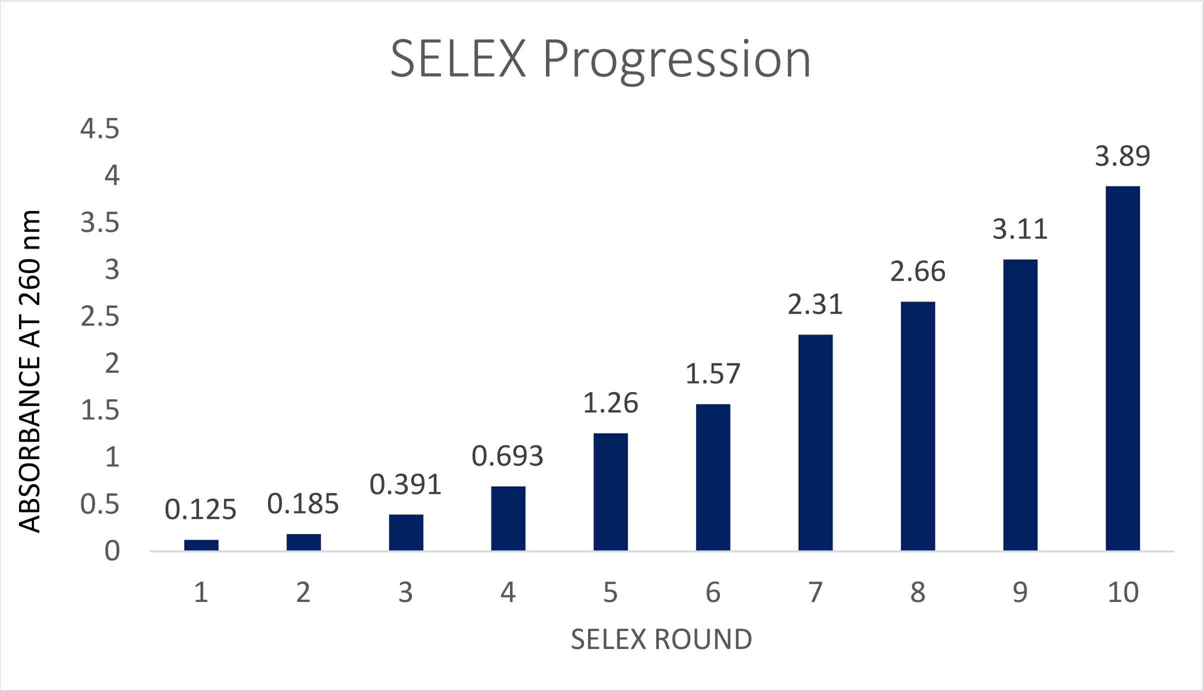

Fig.S1: Graphical representation of SELEX progression

```
CLUSTAL O(1.2.4) multiple sequence alignment


LAP1      GGGCGTTAAAGATACAGGAACCGGTGTCGAGGTC-TGAAGAATCCT----------      45
LAP3      TGGCGTTAGAGATACCGGAACCGGTGTCGGGCGTCTGAAGAATCC-----------      45
LAP2      ---CGT-----ACGTCTGTCTCTGCATCTTATTGCTATGGAATAATGTTGTTCTGG      48
             ***     *     *   * *  **       *   ****
```

Fig.S2 Multiple sequence alignment of the selected aptamers

Sequences identified from 16 clones, aligned by ClustalW. Constant DNA regions are indicated by asterisks at the bottom.

LAP1	LAP2	LAP3
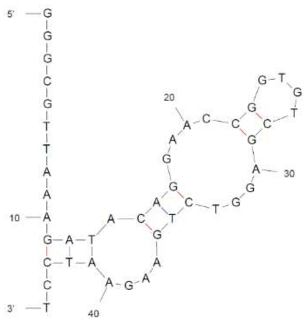	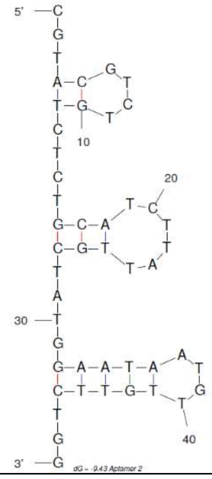	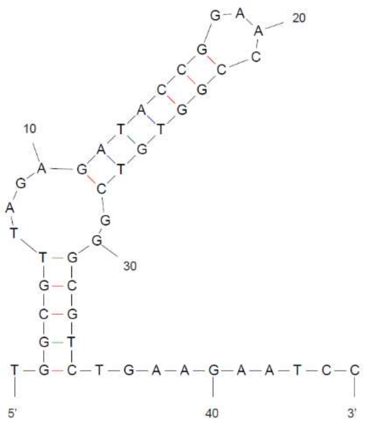
ΔG= -7.47	ΔG= -9.43	ΔG=-13.22

Fig. S3 Secondary Structure prediction of aptamers using Mfold

Predicted possible secondary structures of the aptamer sequences showing the lowest free energy using Mfold software

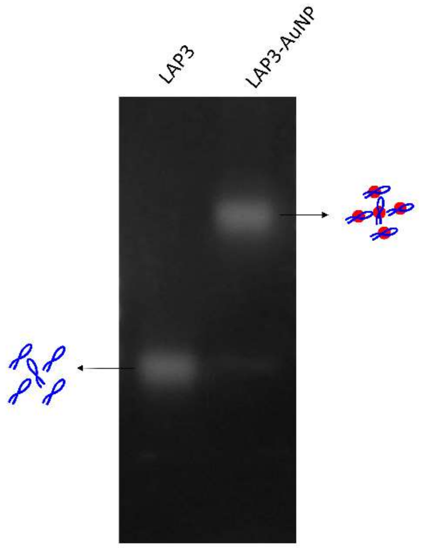

Fig.S4 Electrophoretic mobility of 81bp ssDNA aptamer and 20 nm AuNP-aptamer conjugate on a 2.5% agarose gel.

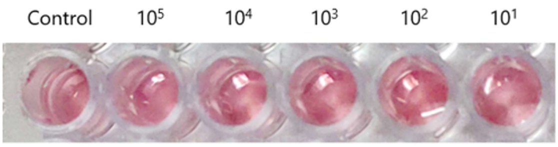

Fig.S5 Effect of different concentrations of *L. interrogans* serovar Autmnalis (6x10^5 to 60 CFU/mL) on AuNPs in the absence of an aptamer

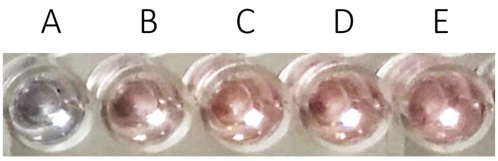

Fig.S6 Specificity of aptamer LAP3

Visible effect on AuNPs, in presence of A: *L.interrogans* serovar Autumnalis, B: *E. coli* C: *P. aeruginosa* D: *S. flexneri* E: *V. cholerae*.

Table 2: Results of water samples screened by PCR and the developed aptasensor.

Sample No	PCR	Aptasensor
S1	-	-
S2	+	+
S3	+	+
S4	-	-
S5	+	-
S6	+	+
S7	+	+
S8	-	-
S9	+	+
S10	+	+
S11	-	-
S12	+	+
S13	+	+
S14	-	-
S15	+	+
S16	-	-
S17	-	-
S18	+	+
S19	-	-
S20	-	-
S21	+	+
S22	+	+
S23	+	+
S24	+	+
S25	+	-
**Total**	16/25	14/25
